# Color standardization and optimization in whole slide imaging

**DOI:** 10.15761/cdp.1000139

**Published:** 2020-09-08

**Authors:** Takashi Inoue, Yukako Yagi

**Affiliations:** 1 Department of Pathology, Memorial Sloan Kettering Cancer Center, 1275 York Avenue, New York, NY 10065, USA; 2 Department of General Thoracic Surgery, Dokkyo Medical University, 880 Kitakobayashi, Mibu, Shimotuga-gun, Tochigi 3210293, Japan

**Keywords:** artificial intelligence, color calibration slide, color standardization, whole slide image

## Abstract

Whole slide imaging (WSI) has various uses, including the development of decision support systems, image analysis, education, conferences, and remote diagnostics. It is also used to develop artificial intelligence using machine learning methods. In the clinical setting, however, many issues have hindered the implementation of WSI. These issues are becoming more important as WSI is gaining wider use in clinical practice, particularly with the implementation of artificial intelligence in pathological diagnosis. One of the most important issues is the standardization of color for WSI, which is an important component of digital pathology. In this paper, we review the major factors of color variation and how to evaluate and modify color variation to establish color standardization. There are five major reasons for color variation, which include specimen thickness, staining, scanner, viewer, and display. Recognizing that the color is not standardized is the first step towards standardization, and it is difficult to ascertain whether the appropriate color of the WSI is displayed at the reviewers’ end.

## Introduction

Color is an integral component of pathology. Pathologists use colored histochemical and immunohistochemical stains to identify structures in the lesion, which aid in rendering a diagnosis. Given the magnitude of a pathological diagnosis in patient management and outcome, it is imperative that pathologists make accurate and reliable conclusions.

Technologies in whole slide images (WSI) have steadily improved in the last two decades [1]. There are now many scanners with faster scanning speeds and higher image resolutions [2]. WSI has various uses, which include primary diagnosis, supplemental information for primary diagnosis, image analysis, education, conferences, and remote diagnostics. It is also used to develop artificial intelligence (AI) using machine learning. In supervised machine learning methods, a few annotations require standardized color for consistency using a display. During this process, the first phase of training data should be of sufficient quality to both assign a correct diagnosis and standardize color to achieve the clinical grade of AI. There are still many aspects to look into critically for implementation in a clinical environment, such as color standardization, consistency of image quality, and system stability. These issues are becoming more important as WSI is gaining popularity in clinical practice.

One of the most important issues is the color standardization of WSI, which emphasizes the importance of color in pathological diagnosis. Poole *et al.* [3] indicated that color-blind pathologists had a lower mean score (94% versus 99%) compared to their colleagues with normal vision when identifying pathological features. Furthermore, Levenson *et al.* [4] demonstrated that pigeons (*Columba livia*) had a lower accuracy in detecting breast cancer using monochrome images compared to full-color images. In the field of color science, it is generally believed that color control is a necessary step in digital imaging, and the U.S. Food and Drug Administration (FDA) [5] has released guidance stating that digital microscope images should be displayed in a consistent and reliable fashion.

The standardization and validation of the color of digital slides on displays is an important aspect of digital pathology. The first workshop to discuss color standardization in medical imaging at the FDA was launched in May 2013, during which there was a discussion on the color standardization of digital microscopy, endoscopy, medical photography, display, and telemedicine. These discussions persist in many fields, such as pathology, endoscopy, and telemedicine.

The effect of color differences in human interpretation of digital pathology images has not been widely studied. However, pathologists themselves have raised concerns that color variability may negatively impact their diagnostic processes. The most common reason for color variation is the differences in the protocols and practices in various histology labs. Additionally, the color displayed can also be affected by a variation in capture parameters such as illumination and filters, image processing, and display factors in the digital systems. These processes are very important and affect one another.

As previously described by Yagi [6], there are five major causes of color variation, which include the thickness of specimen, staining, scanner, viewer, and display. The first step towards standardization is to recognize that the color looked at is not optimized or standardized. No single person is involved in all steps of the process between making a slide and displaying it. Most people are only responsible for one or two processes. For example, a histology technician looks at the physical stain dyes and a stained slide only, the person scanning a slide checks the stained slide and scanned image, and a reviewer looks at the images on their display remotely. The advantage of WSI is that it overcomes the physical distance between the slide and the reviewers. Regardless, it is difficult to ascertain whether the appropriate color of the WSI displays on reviewers’ end, or even on a local display station. Hence, it is very difficult to standardize the color at every point in the entire process, from staining to displaying the scanned slides. Its effects on the final resolution is not well known, although the importance of color standardization have been described by many researchers.

This review aims to identify the five major causes of color variation and propose a simple way to resolve the issue of color standardization for future AI systems To address color standardization, we must recognize that color and image quality issues are commonplace, and then identify the causative factors of the problems in WSI. Hence, it is necessary to develop methodologies to improve the color quality of WSI and make the solutions public.

## Causes of color variation

### Thickness of specimen

**Figure 1.**
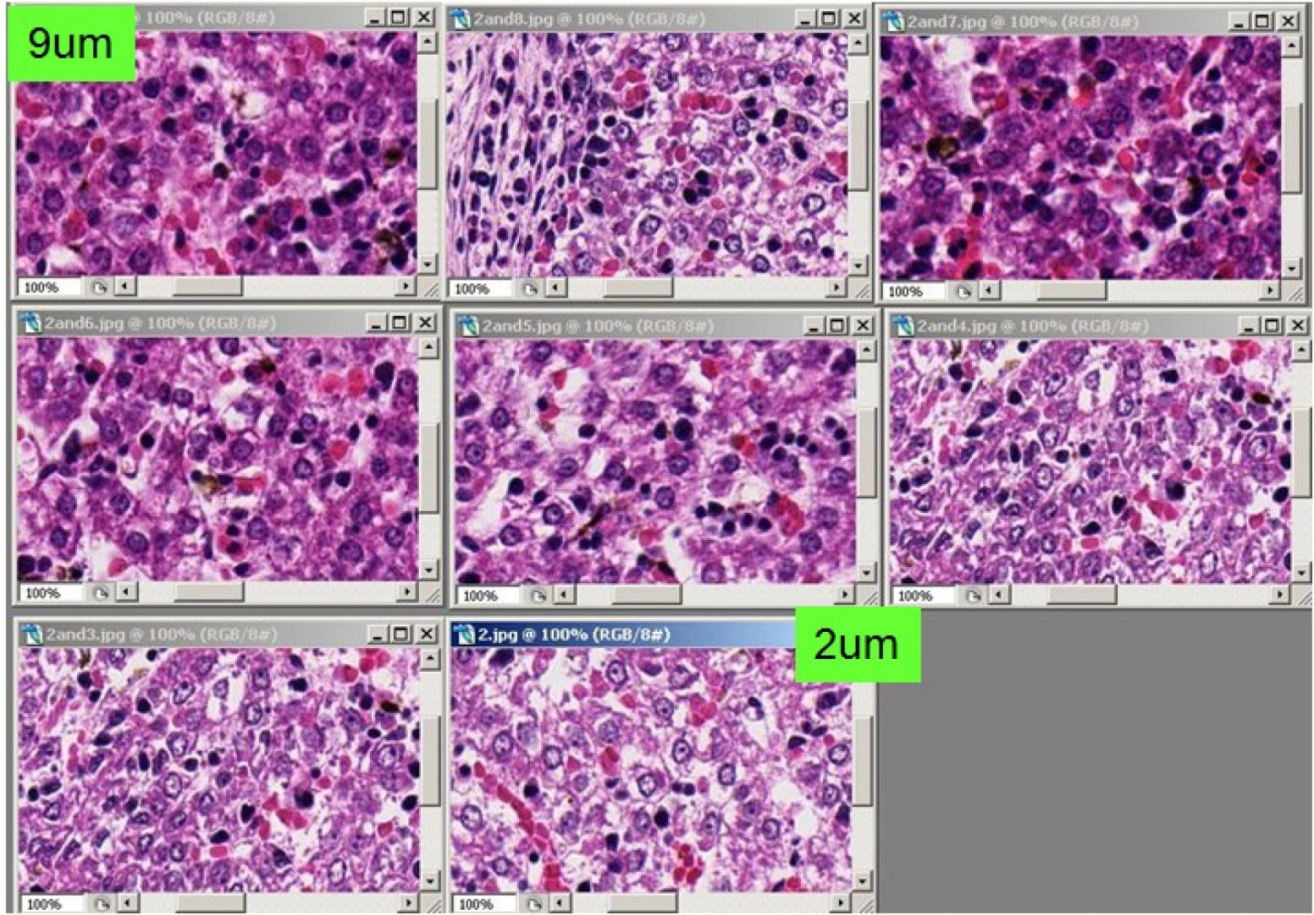
Examples of images made on the same images but of tissues sections cut a various thickness (2–9 μm)

Generally, the thickness of specimen in Japan is 3 – 4 um, and in the United States it is 4 – 5 um. However, this number is targeted and expected thickness rather than measured thickness. The thickness of the tissue section is often not uniform, especially when the tissue size is relatively large. Large tissue sizes may be seen in surgical resection samples, especially in manual sectioning. An automated staining machine is used for Hematoxylin-Eosin (H&E) stain in most major histology laboratories in many countries. Figure 1 shows digitized images of slides in stain dyes, with absorbance by each tissue thickness in the same condition. These tissues were sectioned by an automated sectioning machine to have an adequate consistency in thickness and quality of tissue. The tissues were stained by an automated staining machine at the same time. Thicker tissue slides show darker and unclear details of the tissue, while thinner tissue slides show clearer details in lighter colors. Thus, the thickness of specimen influences the color appearance of the stained slide and scanned image.

### Staining

**Figure 2.**
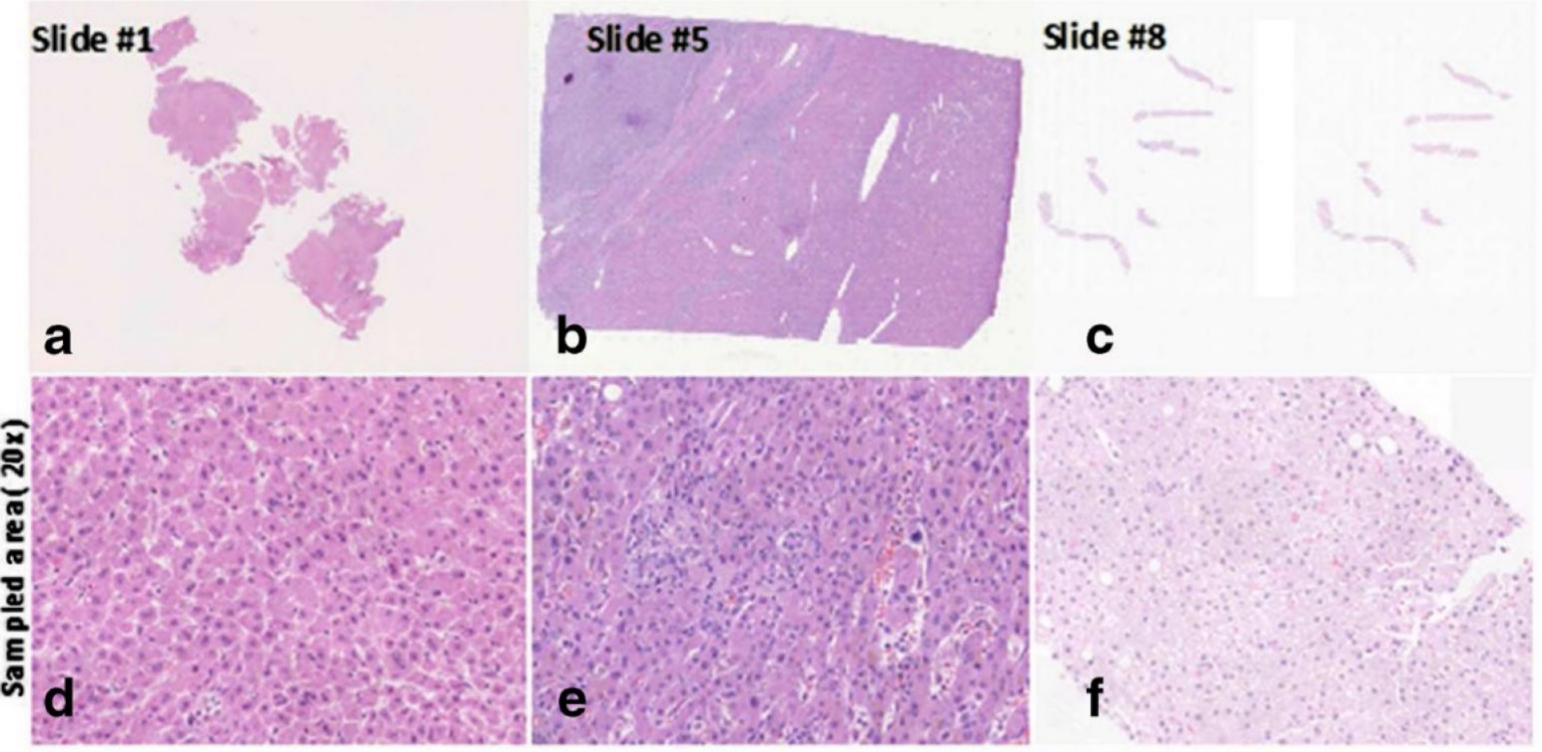
Examples of images that identify the color difference of stain methods (H&E stain)

The appearances of H&E stained slides vary between laboratories or institutions, and between histology technicians. There is a further complication from viewing digitized slides (including WSI) compared to observing the slides under a microscope. This is because the actual stained slides can be seen. Figure 2 shows the color variations of H&E stained slides in different situations. The disparities in routine H&E staining was evaluated by Gray *et al.* [7] who stained the same tissue, scanned it into a digital slide scanner, and performed image deconvolution. While there was high reproducibility in the H&E ratio when staining on the same day (mean difference 0.47%), the H&E ratio varied considerably when stained on different days (mean difference 8.32%). Hence, it must be standardized to the preferred color of each pathologist.

The differences in slide color may have serious implications for the reproducibility of image analysis algorithms. Our recent study, Bautista *et al.* [8] showed that staining tissue affects the results in image analysis. In the study, we classified H&E slides into four classes: nuclei, cytoplasm, red blood cell, and white background. Without any staining correction, the accuracy for each class was 70.626%, 62.279%, 73.399%, and 98.701% respectively. After we conducted staining correction using available tables, the accuracy improved for each class (83.32%, 97.44%, 77.08%, and 99.62% respectively). Additionally, there are numerous papers suggesting algorithms for digital analysis of immunohistochemical stains [9–13]; however, only a few consider the huge implications of color variations in WSI [14,15]. Gavrielides *et al.* [16] reported variation in color between three different WSI instruments from two manufacturers leading to a variation in the performance of image analysis algorithms for HER2 analysis. Approximately 20% to 30% of cases that scored as 2+ on one scanner were re-scored as a different class on another instrument. The staining issue easily affects the difference in training slides such that the results of AI support diagnosis are discordant. There have also been studies about color standardization using software to collect the differences in staining methods, which were discussed by Jain [17] followed by various other studies [14,18–23]. To obtain consistent image analysis results, the appearance of staining should be standardized.

### Scanner and scanning process

**Figure 3.**
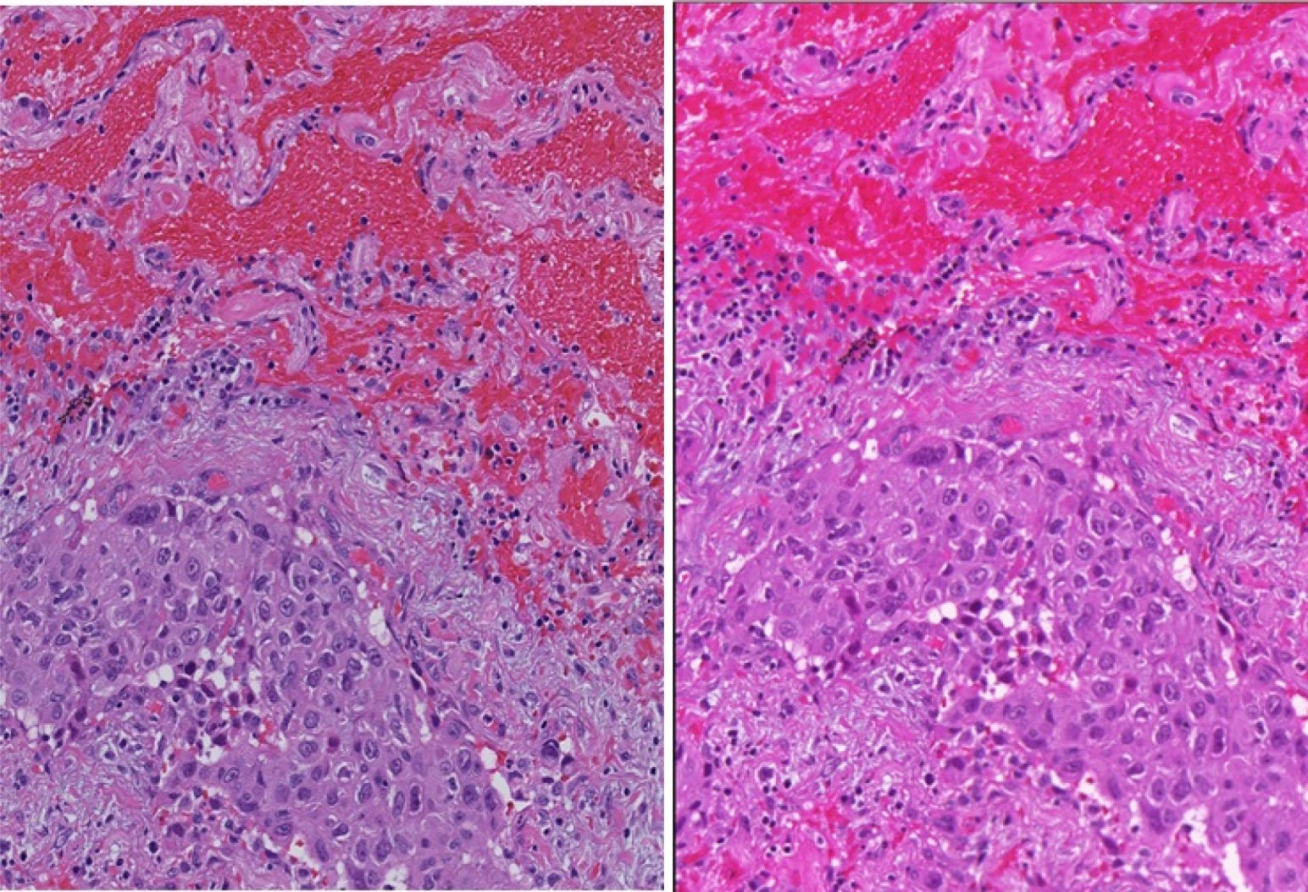
Example of images that identify the color difference of slide scanner

There are many scanners for pathological slides, and every scanner and scanning process produces a different color appearance. A scanner is a combination of many components such as optics, image acquisition device, and image acquisition algorithm. Every vendor has their own private color calibration algorithm, which is the most complicated part in the implementation of color standardization. Figure 3 shows sample images highlighting the color differences between two scanners of the same slide. These color differences also exist between different models from the same company. Creating a different AI system for each machine model wastes time, and the AI system for each model could not address the color difference issue caused by differences between the machines.

### Viewer

**Figure 4.**
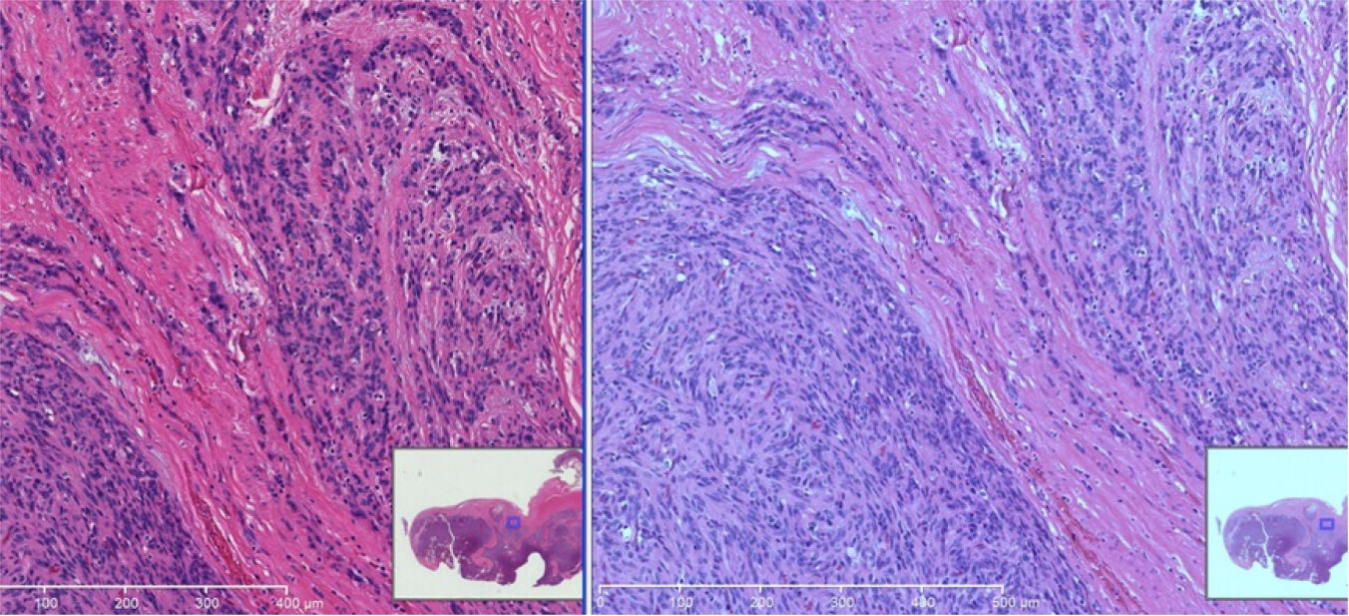
Example of images that identify the color difference of viewer

Similar to scanners, there are also various viewers worldwide. A few of these viewers show multiple WSI produced by other scanners, and it is a very useful function. However, the image quality and color appearance often differ with different viewers. Figure 4 shows an example of the differences seen in various viewers. The original images are the same; however, the image on the right side is brighter than the image on the left. The variations in brightness are due to different color settings of viewer software. It is often difficult for users to ascertain the default setting or accidental setting changes.

### Display

**Figure 5.**
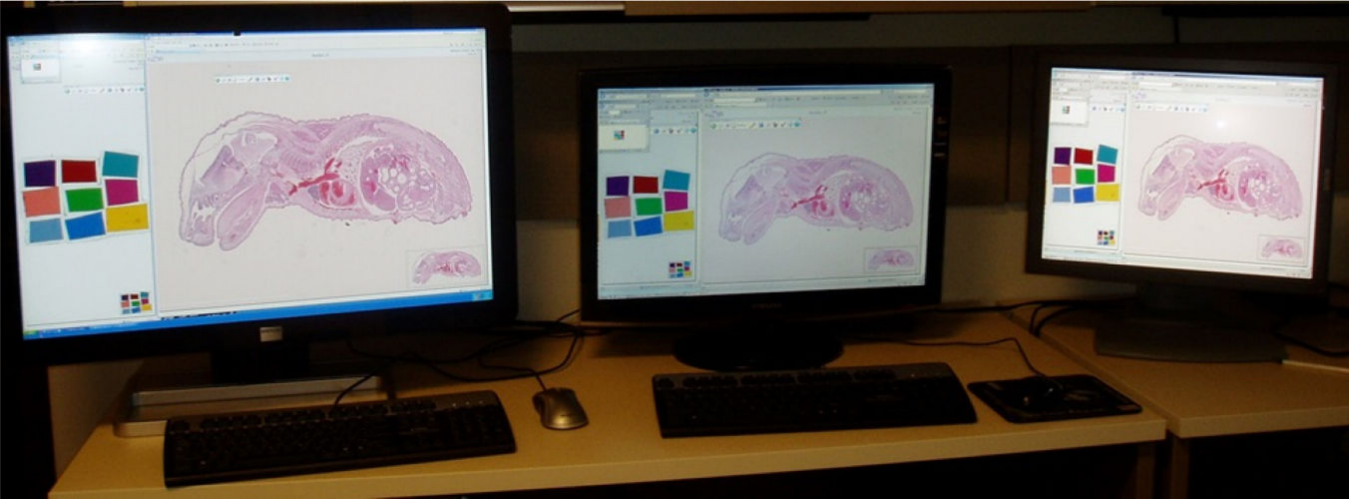
Example of images that identify the color difference of each display

Display is another cause of color variation. Recently, there have been various display types, with different sizes, resolutions, and the capacity to change many settings. Furthermore, there are many variations in the display cards. The matching between the display card, display type, and computer specifications is important to optimally see the original imaging data. However, it is often difficult to attain the best performance of each device. Usually, display settings are inefficiently used, thus most pathologists looking at the same image fail to notice the color differences. Figure 5 shows an example of the differences between three displays. Both the original image and the computer connected to the three displays are the same; however, their colors differ. We conducted a study to determine the differences in color due to the display itself. In the study, we randomly selected 23 standard displays. All driver software and display settings were identical for all 23 displays, and Macbeth color charts was used for standard images. We measured each color on each display with a display analyzer (Display Color Analyzer Model 7123, Chroma ATE Inc).

**Figure 6.**
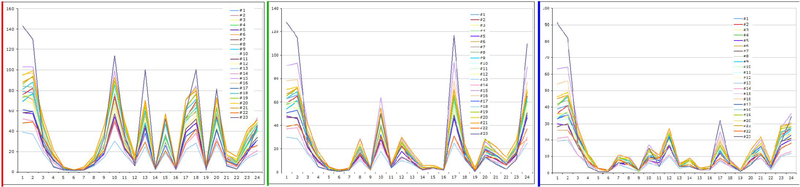
Results of each color component analyzed by digital color analyzer. Figure.6a shows red component, Figure 6b shows green component, and Figure 6c shows blue component

A display analyzer can measure color with three elements: red, green, and blue. Figures 6a–6c show the results of red, green, and blue values, respectively. These figures show that the colors on the displays differ from one display to another, even in same-model displays.

Only a handful of studies have investigated the clinical impact of color standardization and the variation of display characteristics on clinical performance in WSI. Krupinski *et al.* [24] compared a color-calibrated display with an uncalibrated display. They demonstrated that while there was no benefit in color calibration in terms of diagnostic accuracy, there were statistically significant improvements in diagnosis time (mean time to diagnosis calibrated 4.895 s versus uncalibrated 6.304 s, p=0.0046). Similarly, a series of experiments by a vendor, Kimpe *et al.* [25] indicate that color and luminance stability increase diagnostic accuracy and inter-pathologist agreement while also decreasing reading time. However, Hanna *et al.* [26] did not demonstrate an effect of display color standardization on diagnostic accuracy.

## Color standardization

### Color chart

Every user at the imaging facility, including pathologists and engineers, has limited control over the thickness of the specimen, staining process, color of the scanner-produced image, and the viewer. However, if it is possible to know the cause and severity of the acquired inaccuracy, we could optimize the displayed images. Therefore, it is imperative to understand the color variation in a WSI environment to establish a simpler color standardization process. As discussed in our previous study. Bautista *et al.* [27] the staining condition can be controlled and standardized using spectral information instead of RGB data.

Color calibration is an imaging process that seeks to match colors between devices. End-to-end color calibration describes the process of controlling color from source to output through each step of the imaging pathway. Color calibration works by comparing known colors from a set of color patches to the color of those same patches when an image is taken of them with the device. The differences between the known color values for each patch and the color values acquired through imaging allows for a numerical identification of the deficiencies within a specific imaging system. This then allows for the necessary adjustments to be made. Compensation for differences is obtained with an International Color Consortium (ICC) color profile, which can then be used to calibrate and standardize subsequent images. It is important for colored patches to be representative of the colors encountered by the device; otherwise, color standardization may be inaccurate. In photography, digital photographs are often calibrated using a Macbeth color checker, which includes memory colors. Memory colors are patches of critical colors often encountered in photographs, which would result in highly objectionable outcomes if captured incorrectly.

The recent FDA guidance [5] recommends that color standardization should be achieved by using a target slide; The test object should contain a set of measurable and representative color patches similar to the Macbeth color chart, and ideally possess similar spectral characteristics to stained tissue.

### Color calibration slide

**Figure 7.**
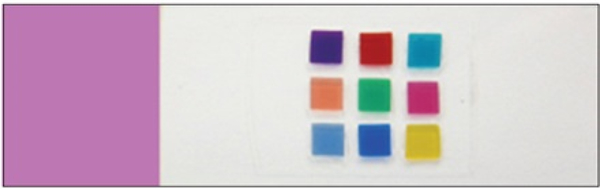
Home-made color calibration slide

The color calibration slide was developed to estimate the color profile of individual scanners and confirm the color calibration of the display. The first color-calibration slide for use in the color correction procedure was made in-house. The calibration slide was made of a typical glass slide embedded with nine color patches. The patches were made of polycarbonate plastic and deep-dyed polyester, and they created colors by allowing only specific wavelengths to pass [28]. For instance, the red color patch absorbed green and blue wavelengths and allowed only the red wavelengths to pass. The mechanism by which these color patches interact with light to achieve their colors is essentially similar to the way stained tissue sections exude their unique colors. Stained tissue sections also absorb light of certain spectral wavelengths and pass the spectral wavelengths associated to their staining. Figure 7 shows a color calibration slide, wherein color patches of sizes approximately 4 mm^2^ are arranged at the center of the glass slide. The patches’ colors include basic colors such as yellow, red, blue, and green, and colors which are generally observed from H&E stained tissue sections [29].

**Figure 8.**
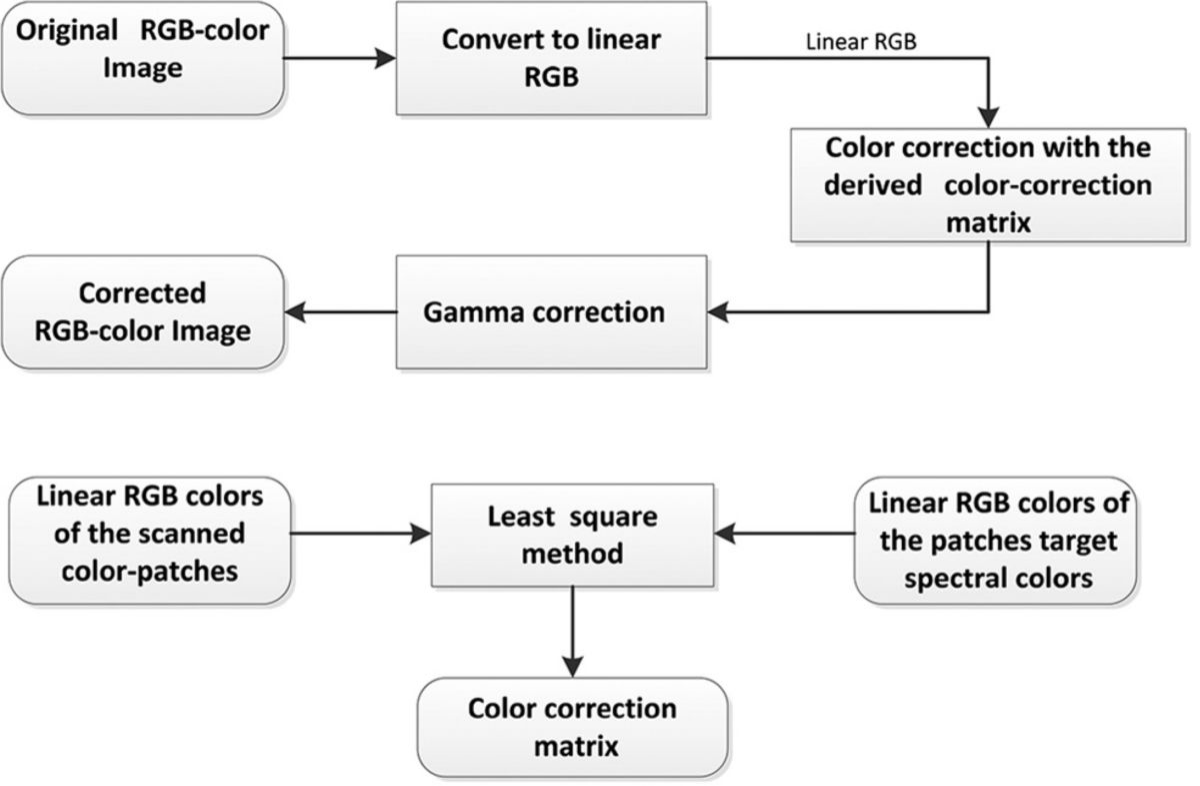
Color-correction scheme process flow; (b) illustration on the derivation of the color-calibration matrix for use in the color correction

**Figure 9.**
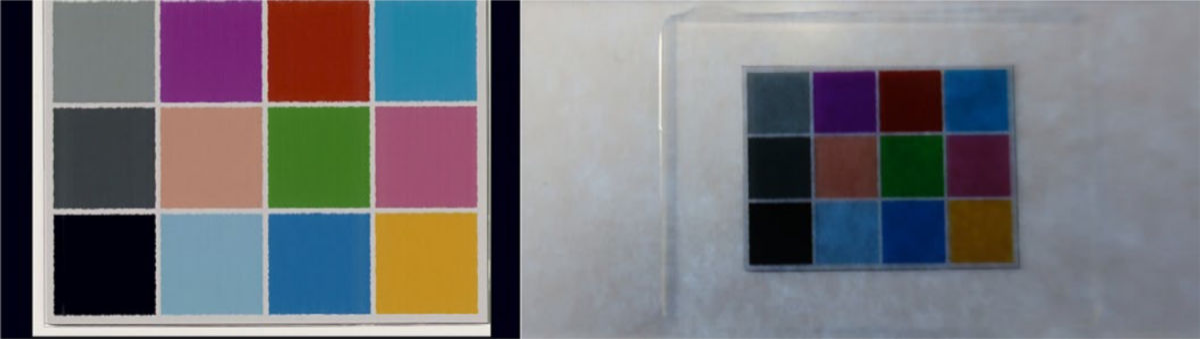
New color calibration chart with 9 color and grey scale (Left). The actual slide with new color calibration chart (Right)

The color combination was selected to best work with H&E staining by multispectral analysis (Bautista and Yagi, 8). The Macbeth color chart is the most common color chart; however, it has twenty-four color blocks, which make it difficult to visually identify color differences. Initially, our slide was designed for a microscope-based imaging system, and all patches were required to be within the field of view of one 4x objective lens and made for accurate color reproduction in pathology imaging, particularly in telepathology (Figure 8). The scanning area of a typical WSI scanner is for the entire glass slide, thus it is easier to create the color chart slide for WSI scanner than for microscopes. As we showed in a former study. Bautista *et al.* [27], the color chart slides with 9 color filters is useful for scanner color standardization. As the slide discussed in our recent study is handmade in the lab, we often found traces of dust and fingerprints on color charts and the cover glass on the slide. Hence, we developed a new color chart slide in collaboration with industries (Figure 9). This further facilitates mass production and the determination of color variations. Additionally, these new slides measured the spectral information of each color patch before being distributed.

The requirements of a calibration slide for WSI are reproducibility, availability of the color information of each patch, low cost, use of the same condition with regular slide such as slide glass and cover glass, and effective automatic scanning by scanners (some of the commercially available charts cannot scan in automatic mode in some scanners). Below, the use of the color calibration slide is described.

### Color correction procedure

Color correction is performed in the CIE XYZ color space [29–31]. This means that space transformation, i.e., XYZ to RGB, is required to visualize the corrected images’ color. We rather adopted the approach proposed in [32] wherein color correction is implemented in the linear RGB color space rather than in the CIE XYZ color space. This could save computational cost when considering the huge file size of WSI. The process flow of the present color correction is illustrated in Figure 8a. The basic mechanism of the color correction matrix is determined in Figure 8b [33].

### Color data of the color patches

#### Target colors of the color patches

The target colors of the color-calibration patches are determined from their representative spectral transmittance samples [34].

#### Scanned colors of the color patches

The scanned colors of the color patches are determined from the average RGB colors of their pixels [35–37].

#### Color transformation matrix

(1)
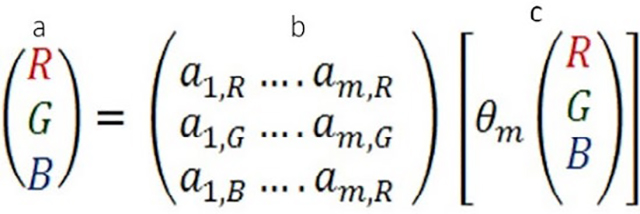


Equation 1 shows the mechanism of color transformation using the calibration slide.

- RGB is the reference color of the color path
- The color transformation matrix is stored for use in color standardization
- The color of the patches as produced by a particular scanner

### Color analysis

The CIELAB color values of the image pixels can be determined in two steps. First, the linear RGB color values of a pixel are mapped unto the CIE XYZ color space using Equation 2. The results of Equation 2 are then used in Eqs. 3 and 4 to determine the corresponding CIELAB color values of the pixel [38]. The variables R, G, and B in Equation 2 correspond to the linear RGB color values of a pixel, and the variables X_0_, Y_0_ and Z_0_, in Equation 3 correspond to the tri-stimulus values of reference white point, which, in our experiments, correspond to white point of the D65 light source.

(2) $$\left[\begin{array}{c} X\\ Y\\ Z \end{array}\right]=\left[\begin{array}{ccc} 0.4124 & 0.3576 & 0.1805\\ 0.2126 & 0.7152 & 0.0722\\ 0.0193 & 0.1192 & 0.9505 \end{array}\right]\left[\begin{array}{c} R\\ G\\ B \end{array}\right]$$

(3) $$\{\begin{array}{c} L*=116f\left(\frac{Y}{Y_{o}}\right)-16\\ a*=500\left[f\left(\frac{X}{X_{o}}\right)-f\left(\frac{Y}{Y_{o}}\right)\right]\\ b*=200\left[f\left(\frac{Y}{Y_{o}}\right)-f\left(\frac{Z}{Z_{o}}\right)\right] \end{array}$$

(4) $$f(x)=\{\begin{array}{cc} x^{1/3} & x>0.00886\\ 7.787x+\frac{16}{116} & x\leq 0.00886 \end{array}$$

The perceptual color difference between two image pixels is proportional to the Euclidian distance between their respective CIELAB color components. The color components in the CIELAB color space are the L^☆^, a^☆^, and b^☆^, where L^☆^ is correlated with brightness, a^☆^ with redness-greenness, and b^☆^ with yellowness-blueness (35). The color difference, dE*_ab_, between two pixels whose respective L^☆^a^☆^b^☆^ values are (L_1_*, a_1_*, b_1_*) and (L_2_*, a_2_*, b_2_*) can be computed using the expression in Equation 5.

(5) $$dE_{ab}^{*}=\sqrt{{\left(L_{1}^{*}-L_{2}^{*}\right)}^{2}+{\left(a_{1}^{*}-a_{2}^{*}\right)}^{2}+{\left(b_{1}^{*}-b_{2}^{*}\right)}^{2}}$$

### Other color standardization slides

There are several studies on color chart slides. Shrestha *et al.* [39] developed a phantom slide with an ICC cross-platform slide that contains 240 colors. However, it is difficult to identify 240 colors manually to correct color variation, and they described only the calibration of their own scanner. Their results do not describe the differences in the viewer and display, thus the final user could not determine the difference from the actual image of origin. Furthermore, Revie *et al.* [40] and Clarke and Treanor [41] developed a calibration slide with nine colors of biopolymer patches that have been stained with pathology staining material. The development makes it is easy to determine color variations within our calibration slide. However, the color patches are products of biomaterial, which means their standard colors will deteriorate in only a few months. Using biomaterial is a suitable method; however, it is difficult for color standardization due to its deterioration effect. Hong *et al.* [42] presented biomaterial color calibration slides for H&E stains only, with promising results. However, these color patches are also biomaterial, thus they are also prone to standard color deterioration within a short time.

### Mouse embryo H&E slide

An ideal protocol should be simple enough to be easily followed, and it is necessary for it to be widely accepted. After the color standardization performed by the color profile calculated from the color chart, the scanning personnel or other users can confirm the standardized color with the mouse embryo H&E stained WSI. The mouse embryo tissue sample contains most of the organ systems even though each organ is still at a tender growth stage. This slide was made for image quality evaluation purposes; however, it is also useful for confirmation purposes. To make similar-conditions slide, 100 slides from one FFPE block were sectioned at once by the automated sectioning system with 5 um thickness and stained with H&E by the automated H&E Steiner. A mouse embryo of about 20 days’ old was selected. All 100 slides were scanned with the same scanner. We recommend using a common slide to easily confirm color standardization.

The color of a WSI can be standardized and confirmed visually by using a color standardization slide, mouse embryo H&E slide, and an application to create a color profile [25].

### Display setting using color standardization slide

**Figure 10.**
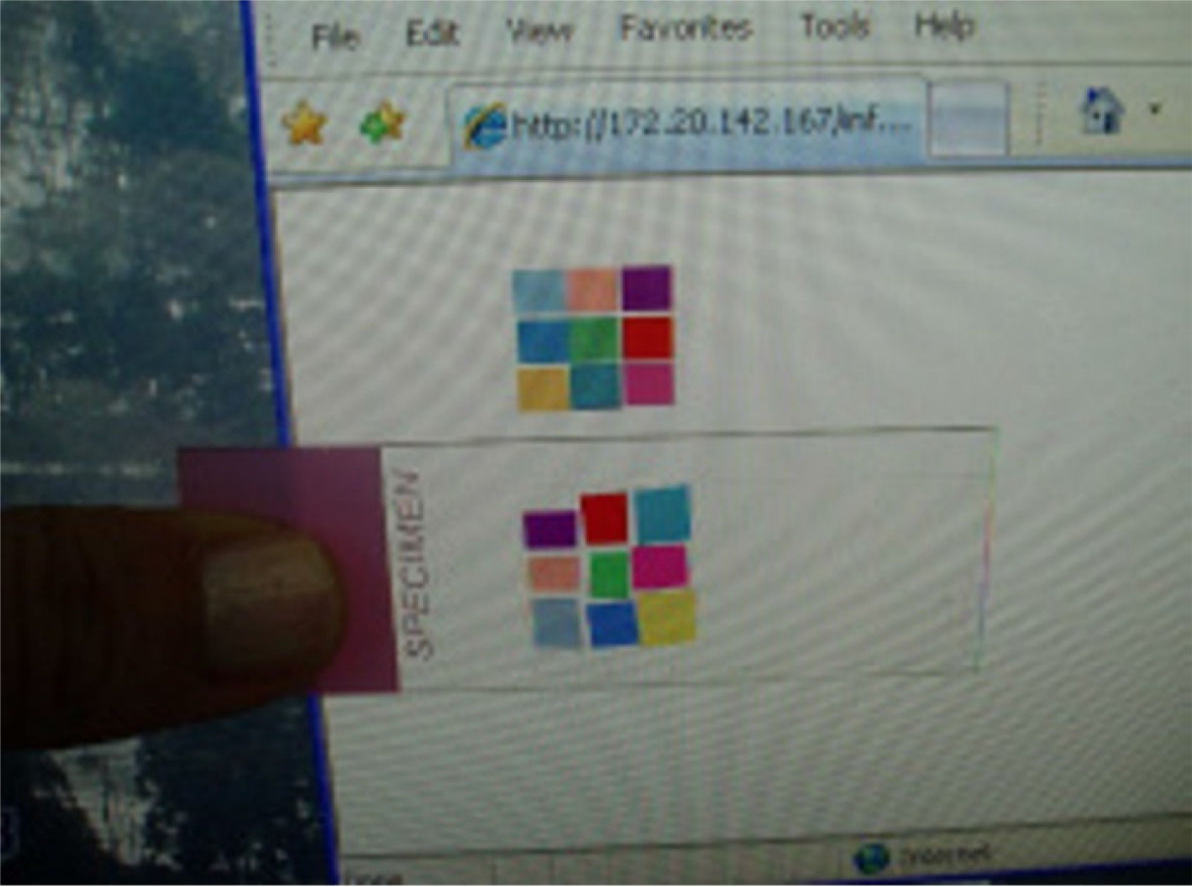
The display color evaluation protocol on how to know whether the color on our displays is acceptable or not. First, a user looks at the calibration web site then checks if all the 9 colors displayed on his/her monitors are differentiated or not. If each color is differentiated, the next step is to compare the color of each patch on the display with the actual slide. The purpose is to know how much the display can produce colors correctly

Finally, pathologists use the display to diagnose digital images. However, as we previously described, there are many color variations due to display issues. To solve this problem, we suggest always using a color standardization slide before using the display system (Figure 10). One should then compare the digitally produced color chart with the physical color chart. If there are any color differences between the physical and display color charts, an effective display calibration tool is commercially available (e.g., Spyder5 Elite, S5EL100, datacolor).

The scanner color profile can be applied to the computer to review images. Recent computer operating systems have color management functions, thus the user can enter the desired color profile. It is also important to note that monitors can degrade with age. In all, we can adjust the color variation issue due to the viewer and display setting.

We have developed, for research purposes, an application to create color profile and then standardize the color between WSI scanners using this new standardization slide. However, we are now developing the mechanism to use it routinely both for research and clinical purposes.

### Guidance and recommendations

As stated previously, the FDA has produced guidance [5] for ensuring color control in digital pathology and also recommended the use of a target slide, ideally with similar spectral characteristics to stained tissue. Furthermore, guidance from the ICC white paper (International Color Consortium, [43] regarding displays for diagnostic digital pathology indicate that all medical-grade displays should be color-calibrated and checked for compliance every 50 days, as the displays can change over time. The white paper also states that ambient light must be stable, given that it is included in the calibration of the display. There are only a few studies that have addressed the topic in digital pathology [24,25], thus there is a lack of primary research on appropriate guidance concerning minimum display requirements for diagnostic digital pathology.

Our method required the targeted color to be standardized. The ideal color or targeted color for standardization is another topic for further research. For example, our target is our standard H&E stained slide at the clinical histology lab.

The FDA-approved scanner for primary diagnosis cannot modify any condition of images.

Our method using color profile will not modify any color of original WSIs. However, it can standardize for purposes such as AI training or a pathologist’s preferences with the viewer side using the color profile associated each WSI. It also requires the data management system to keep the color profile of each image.

### Color

We cannot ascertain whether color and image quality are very important in maintaining high quality WSI, which may vary on a case-by-case basis. In the United States, WSI can be used for clinical diagnosis. In the near future, it is possible that WSI would replace microscope slides even in clinical applications, and there will be an AI system aiding the diagnosis of pathology specimens. To develop a reliable AI system, it is important to give trusted educational material for the machine learning methods. Even small errors in color reproduction, image reconstruction, or analysis results could cause misinterpretation of images. Color standardization is an important aspect in preventing errors in imaging and in developing trusted AI systems and educational materials with digital imaging.

## Conclusion

A pair of calibration slides helps us understand the color standardization problem, and by using the calibration slides we could improve the color of the displays by ourselves. To develop trusted AI systems and educational materials with digital imaging, color standardization is one of the important steps.
